# Body representation in patients after vascular brain injuries

**DOI:** 10.1007/s10339-017-0831-8

**Published:** 2017-08-29

**Authors:** Magdalena Razmus

**Affiliations:** Uniwersytet Marii Curie-Sklodowskiej, Lublin, Poland

**Keywords:** Body representation, Body schema, Body structural description, Body semantics, Vascular brain injury, *Self*

## Abstract

Neuropsychological literature suggests that body representation is a multidimensional concept consisting of various types of representations. Previous studies have demonstrated dissociations between three types of body representation specified by the kind of data and processes, i.e. body schema, body structural description, and body semantics. The aim of the study was to describe the state of body representation in patients after vascular brain injuries and to provide evidence for the different types of body representation. The question about correlations between body representation deficits and neuropsychological dysfunctions was also investigated. Fifty patients after strokes and 50 control individuals participated in the study. They were examined with tasks referring to dynamic representation of body parts positions, topological body map, and lexical and semantic knowledge about the body. Data analysis showed that vascular brain injuries result in deficits of body representation, which may co-occur with cognitive dysfunctions, but the latter are a possible risk factor for body representation deficits rather than sufficient or imperative requisites for them. The study suggests that types of body representation may be separated on the basis not only of their content, but also of their relation with *self*. Principal component analysis revealed three factors, which explained over 66% of results variance. The factors, which may be interpreted as types or dimensions of mental model of a body, represent different degrees of connection with *self*. The results indicate another possibility of body representation types classification, which should be verified in future research.

## Introduction

The human body focuses scientific attention in many disciplines and in interdisciplinary research. As it is the core and material foundation of human existence, it has also become the subject of numerous studies in the field of psychology and neuropsychology (or in broadly conceived neuroscience). Cognitive approach to body experience emphasizes both its complexity and specificity. First, one’s body experience relies on multisensory data integration (Gallagher and Cole 1995; Jeannerod 2004; Holmes and Spence 2004; Giummarra et al. 2008; Medina and Coslett 2010) which result in coherent physical *self* and create sense of body ownership (Haggard et al. 2003; Botvinick 2004; Press et al. 2008; Maselli 2015). Second, body-related data processing involves networks of many brain regions which are spread in the human brain (Churchland 2002). Among them fronto-parietal circuit was evidenced to play predominant role (Rizzolatti et al. 1997; Jeannerod 1999; Galati et al. 2001; Mohr et al. 2006; Tsakiris et al. 2007; Kemmerer and Tranel 2008). Third, human body (faces and other body parts) gains a special status as socially relevant stimuli which play a privileged role in perceptual processing (Bracco and Chiorri 2009; Boyer et al. 2017). As a result, body is a specific mental category within which data are processed faster comparing to other objects of a similar complexity (Shontz and McNish 1972; Ro et al. 2007). Therefore, human minds create and store distinct representations for body and other physical objects (Semenza and Goodglass 1985; Reed and Farah 1995; Coslett 1998; Corradi-Dell’Acqua and Rumiati 2007).

Despite long history and tradition of body representation in psychological and neurological literature (Semenza and Delazer 2003; Vallar and Papagno 2003; Holmes and Spence 2006; Maravita 2006; Gallagher 2006; Corradi-Dell’Acqua and Rumiati 2007; Longo et al. 2010), there is still a lively discussion about its specificity and characteristics. Although results of studies provide evidence that body representation should not be considered as a homogeneous concept but rather as a multidimensional and complex one, with various types to be distinguished, there is still much confusion about these types. Firstly, researchers use many terms and definitions to discuss the types of body representation, and it is not clear to what extent these types are distinct from one another. Such ambiguities result in the usage of manifold body representation measures, which makes the results of studies difficult to compare. Secondly, relations between types of body representation need to be carefully investigated. However, it is evidenced that types of body representation may be dissociated as a consequence of brain injuries (Sirigu et al. 1991; Reed and Farah 1995; Denes et al. 2000; Guariglia et al. 2002; Schwoebel and Coslett 2005; Corradi-Dell’Acqua and Rumiati 2007; Longo et al. 2010); some patients may also reveal deficits in more than one body representation type (Schwoebel and Coslett 2005).

The study by Schwoebel and Coslett (2005) is an attempt to resolve some of the above doubts by investigating types of body representation in patients after brain injuries. Based on recent evidence, the authors assume that there are three types of body representation, which differ in their contents: body schema, body structural description, and body semantics. Body schema is described as a dynamic representation of body part positions, which is derived from sensory data (tactile, visual, vestibular, and proprioceptive) and contributes to the motor system (Schwoebel and Coslett 2005). It gives a basis for the performance of movements by providing information about the dynamic position of each body part in relation to other parts (Sirigu et al. 1991; Denes et al. 2000; Schwoebel and Coslett 2005; Laiacona et al. 2006). As a result, it plays crucial role for body ownership, i.e. the sense that one’s body belongs to oneself (see, for instance, van Stralen et al. 2013; Burin et al. 2015; Llorens et al. 2017). It is created in real time and serves as a basis also for movement imagining (Coslett 1998; Schwoebel et al. 2004; Schwoebel and Coslett 2005; Laiacona et al. 2006). Body schema, similarly to body image, is one of the terms which is the longest used in a scientific reflection on mental models of body. Nonetheless, body schema is diversely defined in philosophical, neurological, and psychological literature and varied relationships between body schema and body image are described. It is noteworthy that some definitions of body schema often do not include postural and dynamic component, but rather emphasize perception of body size and configuration of body parts (Brytek-Matera and Rybicka-Klimczyk 2009) or an ability to experience body boundaries and to locate a stimuli on the surface of one’s own body (see Wolak 1989). In the present study, body schema is understood, following neuropsychological approach, as online and dynamic representation derived from multisensory data which is clearly distinct from map of the body, i.e. body structural description. The latter contains data (mainly visual) about the location of body parts and defines the borders of the body as well as distance relations between body parts (Buxbaum and Coslett 2001; Schwoebel et al. 2004). Body semantics comprises the names of body parts and verbally coded knowledge about body parts (their functions, relations, and associations with objects). Some authors suggest that body semantics also contains information about the typical shape and appearance of body parts (Kemmerer and Tranel 2008). These types of body representation are not only based on various data and processes but also seem to have specific and distinct brain activity patterns. As suggested by Schwoebel’s and Coslett’s study (2005), body image and body structural description are lateralized to the left hemisphere, whereas body schema in not clearly lateralized. Moreover, as the research indicated, body schema deficits were associated with lesions of parietal lobes and/or dorsolateral frontal cortex, whereas form and lexical–semantic knowledge of the body was impaired mainly by temporal lesions (Schwoebel and Coslett 2005).

Systematic group studies on body representation in individuals with brain injuries, such as the research conducted and described by Schwoebel and Coslett (2005), are still rare. Recently, the effects of rubber hand illusion in stroke patients were described (Burin et al. 2015; Llorens et al. 2017), but these reports focus exclusively on reconfiguration of body schema and changes in body ownership. Body representation deficits are analysed mainly in single case studies (Semenza 1988; Chatterjee 1996; Mozaz and Morris 1997; Denes et al. 2000; Buxbaum and Coslett 2001; Guariglia et al. 2002; Felician et al. 2003; Laiacona et al. 2006), which restricts the possibility to drawing more general conclusions on the organization of body representation. There are also doubts connected with the number and content of body representation types as well as with the validity of body representation measures, especially in the case of other concurrent neuropsychological dysfunctions. For this reason and due to the ambiguity of previous conclusions, the current study was undertaken with the following objectives: (1) to describe the consequences of vascular brain injury for body representation and to estimate the prevalence of body representation deficits in patients after strokes; (2) to provide evidence for different types of body representation; (3) to identify the relationship between body representation disorders and other neuropsychological dysfunctions. To accomplish the above aims, 50 patients after strokes and fifty healthy controls were examined with a battery of tasks designed to assess body schema, body structural description and body semantics. As described in “Appendix”, tasks to investigate body schema included hand action and hand laterality tasks. They required motor performance with both hands and motor imagery, respectively. Tasks developed to investigate body structural description involved body parts localization and tactile input localization, both on own body and on the body in a photograph, and matching body parts by location. To assess body semantics, tasks requiring subjects to use semantic and verbally coded body knowledge were applied. They included naming and designating body parts, and matching body parts to objects. In line with the previous literature, it was assumed that brain injury affects body representation and results in deficits of body-related data processing. Since information about the percentage of patients after vascular brain injuries which are impaired on body representation is scarce, a question about frequency of body representation deficits was also posed. As Schwoebel and Coslett (2005) suggest, even more than a half of stroke patients may be affected in at least one body representation measure. In the current study, comparing the scores of patients with these of healthy individuals, the number of stroke patients with body representation deficits was assessed. Further, although the existence of functionally distinct body representation types is suggested in the literature, there is still short evidence considering kinds of body representation components which may be distinguished. To investigate relationships between body representation measures and, as a result, to explore putative types of body representation composed with distinct body-related data, a principal component analysis was performed. Moreover, there have been no clear conclusions considering relationships between body representation deficits and other cognitive dysfunctions. Some of the reports suggest that the former may co-occur with the latter and reflect a more global deficit (see Reed and Farah 1995). On the other hand, some case studies describe patients impaired on body representation measures but intact in other cognitive domains (e.g. Sirigu et al. 1991).

Summing up, it was aimed to make at least three essential contributions with the current study. First, the research is a response to the need for further systematic exploration on the impact of vascular brain injuries on body representation. This is important, as it has been noted, that reports on how often and to what extent strokes impair body representation are not sufficiently discussed in the literature. Second, the research builds on the knowledge on types of body representation, their content, and specificity. It was aimed to investigate structure of body representation in brain-injured individuals with a wide set of tasks considering diversity of body-related data. Third, as there have been little group studies which provide evidence on relationships between body representation deficits and other cognitive dysfunctions, the present study responds to a call for further research in this field. Whereas most prior research has reported results from single case studies, the current one makes an attempt to build on the literature by providing results of a group analysis which sheds light on the deficits co-occurrence.

## Method

### Participants

Two groups participated in the study: patients after vascular brain injuries and healthy controls. All of them were Polish and White.

#### Patients with brain injuries

Because of the specificity of the study, purposive sampling was applied using two main including criteria: vascular brain injury and the patients’ general condition enabling them to sit at the table during the assessment for at least 20 min. There were a number of excluding criteria, namely left-handedness, uncorrected ocular defects or visual deficits, epilepsy, chronic alcohol abuse, cognitive deterioration, dementia, neurodegenerative diseases, past traumatic brain injuries with neurological consequences, and a neuropsychological state that made the examination impossible (e.g. acute confusion, agitation, severe aphasia, or memory impairment). Eventually, the group consisted of 50 participants (24 women and 26 men). Their age ranged from 43 to 85 (*M* = 67.71, SD = 9.65). The mean time that had elapsed from a stroke was 5.35 months (from 1 to 56 months, SD = 10.35), and the mean time spent in the rehabilitation was 22.4 days (from 1 to 73 days, SD = 18.9). The characteristics of the group, based on medical documentation, are presented in Table 1.

**Table 1:** The characteristic of the group of patients after vascular brain injuries (*n* = 50)

	Number of participants	Percentage of participants
Reason of injury		
Ischaemic stroke	47	94
Hemorrhagic stroke	3	6
Lateralization of injury		
Right hemisphere	6	12
Left hemisphere	9	18
Both hemispheres	35	70
Type of injury		
Focal injury	7	14
Diffuse injury	43	86
Paresis		
Left side	30	60
Right side	18	36
Both sides	1	2
Ambulatory capability		
By oneself	21	42
With support	16	32
On a wheelchair	13	26

The mean level of muscle tension in the Lovett’s scale (Cuthbert and Goodheart 2007; Zimmerman-Górska 2012) was 3.33 in the upper limbs (min = 0, max = 5, SD = 1.48) and 3.74 in the lower limbs (min = 1.5; max = 5, SD = 0.93). None of the patients reported body experience dysfunctions, such as somatoparaphrenia, anosognosia, limb personification, kinaesthetic hallucinations, or supernumerary phantom limbs.^1^ One person reported an aversion to a disabled limb (which may be treated as misoplegia). Most of the patients complained about the oddity of an unfit limb, which was sometimes described as “weaker and thicker”, “different than the other one”, “ill at ease”, “dead”, “a pickaxe”, “like not mine”, or “not listening to me”. Most of the patients suffered from other health problems, mainly from cardiovascular diseases (98% of participants), endocrine diseases (58%), and locomotor system diseases (osteoarthritis—24% and osteoporosis—4%).

#### Controls

The group consisted of 50 healthy right-handed adults (27 women and 23 men). The following excluding criteria were applied: cognitive deterioration, dementia, neurodegenerative diseases, past traumatic brain injuries with neurological consequences, stroke, epilepsy, locomotor system injuries and diseases that caused disabilities, not corrected ocular defects or visual deficits, and chronic alcohol abuse. The mean age was 68.17 years (from 43.50 to 85.83, SD = 11.57). A part of this group suffered from cardiovascular diseases (76%), endocrine diseases (22%), and locomotor system diseases (38% had osteoarthritis).

The two groups did not differ significantly in terms of mean age and sex distribution. Patients after vascular brain injuries who participated in the study did not differ from healthy controls in designating objects and in visuo-spatial functions, but they exhibited a significantly lower level of attention, had longer reaction times, and scored lower on naming objects, executing commands, and working memory tasks. All measures used for general neuropsychological assessment are described further in “Measures” section and in "Appendix". For detailed scores and their comparisons between the groups, please see Table 6 in "Appendix".

### Measures

The methods were divided into two groups: (1) methods of general neuropsychological assessment and (2) methods of body representation examination categorized in the three dimensions of body representation: body schema, body structural description, and body semantics.

#### Methods of general neuropsychological assessment

The tasks had a paper-and-pencil as well as electronic form. The set included tasks assessing the following cognitive functions: attention and simple motor reactions, language, working memory, and visuo-spatial functions. All tasks, except for the measure of working memory, were designed by author for the present study. The tasks are described in details in “Appendix”.

#### Methods of body representation examination

The methods of body representation examination were divided into three groups according to the types of body representation (body schema, body structural description, and body semantics). They were designed by the author for the present study and were patterned upon the tasks used by Schwoebel and Coslett (2005). Some modifications were made, both in the number and in the structure of the tasks. All tasks were constructed in order to minimize the usage of verbal communication (when giving the answers) and have a paper-and-pencil or computer form. The stimuli for the body representation tasks were life-like colourful photographs of real body parts and objects displayed on the computer screen and a printed colourful photograph of a human body (A4 size) exposed on the table in front of the subjects. All tasks are elaborated in “Appendix”.

### Procedure

The study had an ex post facto design. The participants were selected by purposive sampling, based on including and excluding criteria (see Participants). All of them gave informed consent. Participation in the study was voluntary, and the respondents did not receive any reward. Before tasks of neuropsychological assessment and body representation were applied, structured interview, observation, and medical data survey were used to receive basic information about the participants and to decide about including them in the study. The interview provided information about subjects’ age, education, handedness, health problems, visual/hearing impairments, self-reliance, previous brain injuries, and drug use. It was also aimed at assessing the participant’s orientation. In the group of patients with brain injuries, questions referring to body experience were also asked. The second part of the interview with participants older than 55 in both groups included the questions of Reisberg’s Global Deterioration Scale (Reisberg et al. 1988; Barcikowska and Bilikiewicz 2004). The observation sheet was used only in the group of patients with vascular brain injuries and included categories such as motor impairments, activity, making contact, communication, and verbal behaviours, following the instructions, reactions and emotional expression, health complaints, untypical behaviours, and other difficulties. In the group of patients, medical data survey was applied. The data were based on medical documentation and considered type and date of the vascular incident, duration of rehabilitation, result of neuroimaging examination, previous brain injuries and their consequences, the patient’s neurological state, self-reliance, and other health problems. The group of participants after vascular brain injuries consisted of patients of the Upper Silesian Rehabilitation Centre in Tarnowskie Góry. They were examined individually, during one, two, or three sessions, depending on a patient’s condition and fatigue. One session lasted an average of 35 min. The assessment of healthy controls was carried out during one meeting that lasted from 40 to 60 min. The measures were ordered in such a way that the tasks which were similar and used the same materials did not occur consecutively.

The study was approved by the Ethics Committee of the Faculty of Pedagogy and Psychology of Maria Curie-Sklodowska University in Lublin.

### Statistical analyses

The following statistics were applied to explore and compare the scores: frequencies, descriptive statistics, the Kolmogorov–Smirnov test, and the independent *t* test and Mann–Whitney *U* test for statistical significance. To examine the structure of body representation in the group of patients after vascular brain injuries, a principal component analysis was applied. Data of 47 participants were taken into account, as three patients were excluded due to missing data. For eight body representation tasks, ten measures were considered, because in two tasks (localizing body parts and tactile input localization) pointing to one’s own body and pointing to the body parts in a photograph were analysed independently. For nine tasks (hand laterality, localizing body parts, tactile input localization, matching body parts by location, naming body parts, designating body parts, and matching body parts to objects), the numbers of correct answers were treated as measures. In hand action task, the number of incorrect movements was computed. For all tasks, standardized results were analysed. To explore the relationships between body representation and cognitive functioning in patients after brain injuries, the co-occurrence of body representation deficits and other neuropsychological deficits with Chi-square test was analysed. It was assumed that a deficit occurred when a patient’s score fell two standard deviations below the mean score of healthy subjects. Additionally, correlations between the results in general neuropsychological assessment tasks and body representation tasks as well as between time since injury and body representation measures were calculated with Pearson and Spearman correlation coefficients.

Data were computed with IBM SPSS Statistics 20.

## Results

### Body representation: intergroup comparisons

Patients after vascular brain injures differed significantly from healthy controls in total scores of all body representation measures. The participants’ results and the comparisons are summarized in Table 2.

**Table 2:** Mean scores of stroke patients and healthy controls in body representation measures

	Patients after brain injuries, *M* (SD)	Healthy controls, *M* (SD)	Mann–Whitney *U*/*t* test	*p*
Hand laterality task—correct answers	24.58 (5.10)	27.00 (4.29)	*U* = 898.00	.05
Hand laterality task—response time^a^ (ms)	4768.36 (3244.06)	3129.38 (1364.24)	*t*(65.81) = 3.29 *d* = 0.63	.001
Hand action task—errors in total	4.12 (4.45)	0.16 (0.62)	410.50	.001
Hand action task—errors for opened eyes	1.86 (2.25)	0.14 (0.45)	607.50	.001
Hand action task—errors for closed eyes	2.18 (2.30)	0.08 (0.40)	438.00	.001
Hand action task—errors for R hand	1.98 (2.47)	0.10 (0.42)	519.50	.001
Hand action task—errors for L hand	2.51 (2.63)	0.12 (0.44)	406.00	.001
Hand action task—performance time^b^ (s) R hand	3.12 (2.24)	1.09 (0.35)	*t*(41.59) = 5.76 *d* = 1.11	.001
Hand action task—performance time (s) L hand	3.40 (2.09)	1.12 (0.35)	*t*(46.19) = 7.22 *d* = 1.23	.001
Localizing body parts—correct answers for own body	15.84 (1.46)	16.70 (0.61)	*U* = 788.50	.001
Localizing body parts—correct answers for the photograph	15.96 (1.21)	16.70 (0.61)	*U* = 788.50	.001
Tactile input localization task—correct answers for own body	21.71 (0.62)	22.00 (0.00)	*U* = 950.00	.001
Tactile input localization task—correct answers for the photograph	21.77 (0.56)	22.00 (0.00)	*U* = 1000.00	.01
Matching body parts by location—correct answers	10.73 (2.29)	12.08 (1.37)	*U* = 760.50	.001
Matching body parts to objects—correct answers	9.54 (0.85)	9.96 (0.20)	*U* = 893.00	.001
Naming body parts—errors	5.52 (4.06)	1.82 (1.70)	*t*(62.45) = 5.85 *d* = 1.03	.001
Designating body parts—correct answers	21.56 (1.21)	22.00 (0.00)	*U* = 1050.00	.01

*L* left, *ms* milliseconds, *R* right, *s* seconds

^a^Patients needed significantly more time to give correct answers for each hand and in each placement (see Appendix, Table 7)

^b^Statistically significant differences were also found between the groups in the mean time of movement performance for both types of conditions (see Appendix, Table 8)

### Body representation in patients after brain injuries: intragroup analyses

#### Time since injury and body representation

Median split on time since injury was used to divide subjects into two groups (Me = 2.01 months) to explore whether the time may affect results in body representation measures. Comparisons revealed that there were no statistically significant differences in body representation tasks between patients who experienced stroke less than 2 months and more than 2 months before the assessment. Furthermore, in the sample there were no significant correlations between time since injury and body representation scores.

#### Principal component analysis

The appropriateness of component analysis for the observed correlations was suggested by the value of .70 resulting from the Keiser–Meyer–Olkin measure of sampling adequacy. Examination suggested three components, which accounted for 66.55% of the variance in the results. Component loadings are presented in Table 3.

**Table 3:** Component loadings (C1–C3) and the percentage of variance accounted for principal component analysis with varimax rotation

Task	C1	C2	C3
Localizing body parts in a photograph	**.882**	−.097	.101
Localizing body parts on own body	**.830**	−.006	.075
Matching body parts by location	**.778**	.121	.106
Designating body parts	**.769**	.164	−.070
Matching body parts to objects	**.715**	.119	.418
Naming body parts	**.671**	.368	.246
Tactile input localization on own body	−.021	**.886**	.005
Hand laterality task	.180	**.778**	.126
Hand action task	.016	.043	**−.848**
Tactile input localization in a photograph	.218	.183	**.568**
Per cent variance	37.04	16.26	13.25

Numbers in bold indicate strong component loadings

The results of principal component analysis and their possible explanations will be discussed further in “Discussion” section.

#### Body representation deficits

Subsequently, it was checked whether there were deficits in single body representation tasks in patients after brain injuries. Performance of the patients was compared to the performance of healthy individuals. The scores that were at least two standard deviations below the mean score of healthy controls were considered as an indicator of a deficit in the task. Interestingly, only nine out of 47 patients (19%) were not impaired in any of the ten body representation measures. Data about deficits in body representation measures are presented in Table 4.

**Table 4:** Number and percentage of patients with deficits in body representation measures (*n* = 47)

Task	*n* (%)
Hand action task	28 (60)
Naming body parts	20 (42)
Matching body parts by location	19 (40)
Localizing body parts on own body	14 (30)
Localizing body parts in a photograph	14 (30)
Matching body parts to objects	14 (30)
Tactile input localization on own body	10 (21)
Tactile input localization in a photograph	8 (17)
Designating body parts	8 (17)
Hand laterality task	7 (15)

#### Relationships between body representation deficits and neuropsychological variables

Ten patients had neuropsychological deficits without body representation deficits. In four participants after brain injuries, the pattern of deficits was opposite: there were a deficit in at least one body representation measure but no other neuropsychological deficits. Analysis with Chi-square test revealed that there existed statistically significant relationships between some deficits. Chi-square test values for these relationships are presented in Table 5.

**Table 5:** Chi-square values for the significant relationships between neuropsychological deficits and body representation deficits

Neuropsychological deficit	Deficit in body representation task	Chi-square values	*p*
Deficit of working memory	Hand laterality task	8.04 *φ* = 0.52	.01
	Tactile input localization on own body	4.60 *φ* = 0.40	.05
	Designating body parts	6.60 *φ* = 0.47	.05
Deficit of visuo-spatial functions	Localizing body parts on own body	9.67 *φ* = 0.50	.01
	Localizing body parts in a photograph	14.52 *φ* = 0.60	.001
	Matching body parts by location	7.61 *φ* = 0.40	.01
	Matching body parts to objects	5.26 *φ* = 0.40	.01
Deficit of naming objects	Naming body parts	5.60 *φ* = 0.33	.05

For these statistically significant relationships, correlation coefficients between total scores in the tasks were computed. As analyses showed, only two correlations were statistically significant: between scores in the visuo-spatial functions task and in the matching body parts by location task (*ρ* = .64, *p* < .01) and between scores in the visuo-spatial functions task and the task of matching body parts to objects (*ρ* = .48, *p* < .01).

## Discussion

The main aim of the study was to describe the state and structure of body representation in patients after vascular brain injuries. The results obtained by patients and healthy participants in tasks designed to measure various types of body representation were computed and analysed. The study was based on the work of Schwoebel and Coslett (2005), which, to the best of my knowledge, is the only study on various types of body representation in a large group of participants after brain injuries. It was planned to test the model that distinguishes three types of body representation: body schema, body structural description, and body semantics, as well as to compare the results to those obtained by Schwoebel and Coslett in their study.

Firstly, intergroup comparisons revealed that vascular brain injury results in body representation disintegration, which is manifested in deficits of: (1) movement planning, movement performance, and movement control for both hands; (2) imagining the movements of one’s body and performing its mental rotation; (3) the ability to localize body parts and to apprehend spatial relationships between them; (4) designating and naming body parts, as well as deficits in semantic knowledge about body parts, including the associations of body parts with objects. Because there is still a scarcity of studies on body representation in patients after brain injuries, and because epidemiological data on body representation deficits in this group are still rare and remain inconsistent, the question about the frequency of body representation deficits was valid. Deficit in at least one body representation task appeared in 81% of patients after bran injuries. The rate is much higher than the 51% found in Schwoebel and Coslett’s study (2005). It can therefore be concluded that some kind of body representation deficit is a common consequence of brain injury. In the present study, this referred the most often to dynamic body representation, which enables a person to perform previously planned movements. This deficit manifests itself in difficulties in moving one’s hand on command (in 60% of patients). The result remains in line with the research of Llorens et al. (2017), which indicates that stroke is a common cause of body schema disorder, influenced by motor impairment and a suppression of the reflex activity. In more than a half of the patients, deficits involved naming body parts and matching body parts by location. The least frequently observed deficits (in 25% of patients) concerned tactile input localization, designating body parts, and determining the side of a hand (in hand laterality task). Data showing the infrequent prevalence of impairments in understanding terms describing body parts are consistent with previous reports. It was proven that some aspects of body representation (e.g. designating body parts) are more resistant to distortions resulting from brain injury (Kemmerer and Tranel 2008). There were no correlations between time since injury and body representation state.

Secondly, the results of principal component analysis suggest that it is possible to speak about functionally distinct types or components of body representation, which is in line with the results of previous studies of patients after brain injuries (both group and single case studies) (Sirigu et al. 1991; Coslett 1998; Buxbaum and Coslett 2001; Schwoebel and Coslett 2005). However, the results of the analysis do not confirm the components that were obtained by Schwoebel and Coslett (2005) in 70 stroke patients. Principal component analysis suggests that three components can be distinguished. The first one is loaded by tasks which, according to Schwoebel’s and Coslett’s assumptions, are elements of both body structural description (localizing body parts and matching body parts by location) and body semantics (designating body parts, naming body parts, and matching body parts to objects). When trying to explain and interpret the first factor, one should note that all tasks which load on this component can be characterised by a significant share of the semantic factor. Tasks connected with body semantics require the use of body parts names and the use of knowledge about the associations between body parts and objects (clothes or accessories). Tasks which are usually treated as body structural description measures are also based on concepts and their categorization, as they require the use of semantic elements of body representation, such as verbally coded knowledge about the appearance, shapes, and locations of body parts (Kemmerer and Tranel 2008). Moreover, in the task of matching body parts by location, a participant probably categorizes body parts in terms of body region first (eliminating parts from a different body area) and only later analyses the spatial relationships between body parts. Similarly, pointing to body parts on one’s own body and on a body in a picture presumably requires the use of body part concepts. Furthermore, when explaining the first component, it is important to remark that all tasks which load on this component use specific material, namely photographs of isolated body parts. Such material requires references to single parts only, without the need to analyse the body as a whole. What is more, isolated body parts are neutral elements of general body representation that are not directly connected with the *self*. It can therefore be stated that tasks which use pictures of isolated body parts and load on the first factor involve the processing of data about the body on a kind of “impersonal” level. The second component is loaded by the tactile input localization task (a measure of body structural description in Schwoebel’s and Coslett’s study) and the hand laterality task (a measure of body schema according to these authors). The first task consists in feeling touch and mapping the input onto body surface, and the latter requires feeling one’s own-body position and performing mental rotations of body parts. It can be concluded that both tasks involve the representation of one’s own body as well as basic body experience. The third component is loaded by two tasks. One is treated as a measure of body schema (the hand action task) and the other as a measure of body structural description (tactile input localization in a photograph). It is noteworthy that the two tasks involve both the representation of one’s own body (movement performance and localization of touch) and a mental model of another person’s body (the investigator’s body or a body presented in a picture). Therefore, they both require a “translation” or “conversion” between “body-as-an-object representation” and “own-body representation” (observed movement vs. one’s own motor programme in the hand action task, and felt touch vs. input localization on another person’s body in the tactile input localization task).

The factor structure of body representation in patients after vascular brain injuries in the present research diverges from the structure described by Schwoebel and Coslett (2005). The difficulty in comparing the results of both studies directly may be a consequence of methodological solutions, e.g. different tasks which were used in the studies. Furthermore, the sample of patients in the current research differed from that of Schwoebel and Coslett (2005) in terms of number of participants, their age, and brain injury location. In the current project, the sample was smaller, the average age of participants was higher, and location of post-stroke injuries was not confined to a single hemisphere which may have contributed to some discrepancies in the results. On the other hand, the findings may initially indicate that components can be marked off not only on the basis of their contents, which is connected with processes involved (motor, visuo-spatial, lexical, and semantic), but presumably also on the basis of their relationship with the *self*. Body representation seems to consist of various components or elements (or types), which are “saturated” with the *self* to various degrees. Interpreting the obtained components, I suggest that the first component is the least strongly connected with the *self* and relates primarily to body as an object, which is processed on an “impersonal” level. This component is associated with “Me” as an object of experience and reflective awareness (Tagini and Raffone 2010). The relationship with the *self* in the second component is the strongest—the tasks require the processing of sensations that come from one’s own body and are connected with the basic aspects of body experience. Their performance demands updating of the relative positions of one’s body parts (a kind of body map) using both static and dynamic proprioceptive signals, and knowledge of the position of one’s body to programme and guide direct actions towards the body (Paillard 1999; Anema et al. 2009). The component is strongly related to “I” or subjective sense of the *self* where the body itself is a subject of experience and the first-person perspective is taken (Tagini and Raffone 2010). The third component relates both to the body as part of the *self* and to representations of other bodies (body as an element of physical and social environment). It comprises the processes of comparing the *self* with *others* and conversion between the two aspects of the mental model of a body.

Apart from the abovementioned differences, there is also a similarity between the factor structure of body representation in Schwoebel’s and Coslett’s study and the one described in this text. In both studies, the hand laterality task and the hand action task, which are assumed to measure body schema, loaded on two separate factors (in Schwoebel’s and Coslett’s study each of the mentioned tasks loaded on a distinct factor, which was not loaded by any other task—that is, the factors were loaded by one task only; in my study, the tasks were included in diverse factors, which were also loaded by other tasks). This kind of separateness of these two tasks is inconsistent with the findings reported in the literature, which indicate that both tasks refer in a similar way to dynamic body representation (Parsons 1987; Stephan et al. 1995; Coslett 1998; Jeannerod 1999; Gerardin et al. 2000; Schwoebel et al. 2001, 2002; Amorim et al. 2006; Sekiyama 2006; Creem-Regehr et al. 2007). This issue needs to be explored both in clinical samples and in healthy participants.

Summarizing the results concerning the relationships between body representation and general neuropsychological functioning, it is legitimate to conclude that in a considerable proportion of participants with vascular brain injuries deficits in body representation tasks co-occurred with neuropsychological deficits but only several relationships between the two types of deficits appeared to be statistically significant. There were associations between three body representation tasks (measuring tactile input localization on one’s own body, designating body parts, and determining the side of the hand) and working memory deficit. Furthermore, visuo-spatial deficit was related to performance in the tasks of matching body parts by location, matching body parts to objects, and localizing body parts. Finally, deficit in naming objects was significantly related to deficit in naming body parts. It should be emphasized that in the group of patients with deficits there were only two significant correlations between scores in body representation tasks and general neuropsychological assessment tasks (it referred to visuo-spatial processing and matching body parts by location, as well as visuo-spatial processing and matching body parts to objects). The results suggest that only for the abovementioned pairs one can conclude on the correlations between the level of body representation deficits and cognitive dysfunctions. The extent of distortions in other aspects of body representation does not have to be related to the intensity of concurrent neuropsychological dysfunctions. On the other hand, there were also some participants after brain injuries who exhibited cognitive dysfunctions without body representation distortions, as well as those in whom, conversely, body representation deficits were observed without co-occurring neuropsychological dysfunctions. There were no associations between deficits in attention, executing commands, and designating objects and deficits in body representation tasks. The results of the present study suggest that body representation deficits may co-occur with cognitive deficits, which is in line with the results of previous research (Semenza and Goodglass 1985; Reed and Farah 1995; Denes et al. 2000; Laiacona et al. 2006; Corradi-Dell’Acqua and Rumiati 2007; Kemmerer and Tranel 2008), with the reservation that the latter are a background or possible risk factor for body representation deficits rather than sufficient or imperative requisites for body representation deficits. Moreover, the extent of body representation impairment does not have to be related to the extent of concurrent neuropsychological dysfunctions. Such conclusions are consistent with the observations concerning patients reported in the literature and showing that impairments of body representation with intact functioning of other cognitive domains, as well as reverse situations, are possible (Semenza and Goodglass 1985; Semenza 1988; Suzuki et al. 1997; Guariglia et al. 2002).

To sum up, results of the present study contribute to expand the knowledge about how body representation is affected by vascular brain injuries. It was evidenced that strokes often result in body representation disintegration, which is observed in deficits in various body representation measures. These deficits may be related to other cognitive dysfunctions, but may be also dissociated and independent from them. Moreover, body representation revealed in patients after vascular brain injuries its multifaceted structure, within which distinct types of body representation may be distinguished. The conclusions discussed, particularly the interpretations of body representation components which might be diversely related to the *self*, should be treated as suggestions which may outline directions for future research. The proposal should be verified and the relationships between body representation and the nature and functioning of *self* should be explored. Addressing these questions in future studies may provide some advances in the field of associations between various facets of consciousness and self-related processes which still remain uncertain (Tagini and Raffone 2010).

Despite the contribution the presented study makes to the knowledge on body representation, it has its limitations. First, the research groups were not very large. It would be interesting to verify the results in more numerous samples. Second, the group of patients after vascular brain injuries was diverse in terms of age, time which passed from the injury, and brain injury locations. Future studies including more homogenous samples would be beneficial for clarifying the question of body representation structure. Third, methods with unknown validity were used mostly in the study due to specific aims of the research. It would be advisable to investigate psychometric properties of the methods and verify them in further studies. Last, the heterogeneity of the sample in terms of brain injury location and limited information about the location in some cases made it impossible to draw the conclusions on probable brain correlates of the distinguished body representation types.

## Appendix: Measures

### Methods of general neuropsychological assessment

#### Attention and simple motor reactions

The participant’s task is to press a button on a modified computer keyboard when a specified sign (a star) is displayed on the screen. Participants are asked to look at the centre of the screen and to react immediately after the target sign appears. The target sign and distractors are displayed singly (on the left or right side of the screen) and paired with other signs (also presented on both sides of the screen). The scored part of the task is preceded by a training. In the scored part, the target sign is exposed 12 times: six times singly (three times on the left and three times on the right side of the screen) and six times in pairs (three times on the left and three times on the right side of the screen). The distractors appear nine times (six times singly and three times in pairs with other distractors). The exposition time is 6 s. The number of correct answers and reaction time are measured.

#### Language

In the comprehension task, the participant is asked to point on the computer screen to an object which is named by the experimenter. Sets of five or six objects appear on the screen. A total of 35 objects are used, divided into five categories: fruits and vegetables, animals, pieces of furniture, utensils, and other accessories. The number of correct answers is measured.

In the naming task, the participant is supposed to give the name of the object that is displayed individually on the computer screen. The task applies the same photographs that are used in the comprehension task described above. The number of correct answers and errors are measured.

The execution of commands task is based on a similar task from the Boston Aphasia test (Kaplan et al. 1983) and comprises five commands of increasing difficulty. The experimenter reads a command, which may be entirely repeated if needed. The score in the task is the number of correctly performed parts of each command (e.g. the command *Please*, *point to a window and then to a ceiling* is scored 2 points, as it contains two parts). The maximum score is 15.

#### Working memory

To assess a participant’s memory, Digit Span subscale from Polish adaptation of WAIS-R was used. The task was performed according to the standardized procedure (Brzeziński et al. 2004).

#### Visuo-spatial functions

The idea of the task comes from Ogden’s work (1985). A printed colourful photograph of a bicycle (A4 format) is presented at the table to the participant. Colourful pictures of nine bicycle parts (e.g. wheel, rack, saddle, and pedal) are displayed individually on the computer screen. The participant should point, in the printed picture, to the part of a bicycle that is visible on the screen. The number of correct indications is measured.

### Methods of body representation examination

#### Body schema tasks

Two tasks were used to examine dynamic body representation: a hand laterality task and a hand action task.

In the hand laterality task, the participant is asked to decide whether the hand in the photograph displayed on the computer screen is right or left hand. Prior to the task, to make sure that the participant is able to comprehend the instruction, he/she is asked to point to his/her left and right hand. The pictures of left and right hands placed palm-up and palm-down are displayed in a random order in four rotations: fingers pointing away, fingers pointing towards the subject, fingers pointing to the right, and fingers pointing to the left. To give an answer, the participant is asked to press one of the two buttons (R for right or L for left) on the modified keyboard with his/her able hand. A short training with five photographs precedes the assessment stage. At the assessment stage, 32 expositions are used, since each hand in all placements and rotations is exposed twice (eight photographs of the left hand—four palm-up and four palm-down, in four rotations; eight photographs of the right hand—four palm-up and four palm-down, in four rotations). Participants are asked to keep their both hands in the same palm-down positions (the hand used to press the button lies between R and L button, while the other one should take the palm-down position on the table or on the participant’s thigh). There is no possibility to move any of one’s hands to make the task easier. The task is not time limited, and it is possible to come back to a previous picture and change the answer. The number of correct answers and response time are measured by the computer programme. It should be noted that some modifications were made to the version of the hand laterality task used by Schwoebel and Coslett (2005). Firstly, the current version is based on colourful pictures displayed on the computer screen. Secondly, fewer expositions are applied in it (32 instead of 64). Thirdly, to give an answer, participants use the modified keyboard instead of moving their own hand as in Schwoebel’s and Coslett’s study. Lastly, the position of participants’ hands is controlled by keeping them palms down.

The hand action task consists of four movements of the hand and fingers, which are demonstrated by the investigator (clenching the fist, touching the last finger with the thumb, touching the thumb with both the index finger and the middle finger, and putting the thumb between the index finger and the middle finger). The participant should repeat every movement three times as quickly and as accurately as possible with both hands independently (first with the right hand and then the left hand). Afterwards, the same movements are performed with eyes shut (again, three times for each movement, with both hands independently). Patients with severe hemiparesis or paralysis perform the movements only with the intact hand. The participant starts each trial after a verbal signal given by the experimenter. The time of each movement is recorded with a stopwatch. The number of incorrect movements (in total and for each hand in both types of conditions) as well as time of movement performance is measured. An error is noted when the arrangement of the finger(s) is incorrect or when a movement is repeated an incorrect number of times (e.g. when perseveration occurs). An error is not noted when a participant is unable to perform a movement (in such situations, the participant’s result should be excluded from the analysis). Modifications to the original task used by Schwoebel and Coslett consisted in reducing the number of movement repetitions. Moreover, the imagery part of the task (in which the participant is asked to imagine the movements) was replaced by performing the movements with one’s eyes shut.

#### Body structural description tasks

Three tasks were used to assess body structural description: body parts localization, tactile input localization, and matching body parts by location.

In the body parts localization task, participants see 17 colourful photographs of isolated body parts on the computer screen (hand, mouth, abdomen, knee, forehead, arm, brow, thumb, thigh, ear, elbow, foot, chest, calf, ear, neck, and eye). The task is to point to the same part on their own body and then on the body in the colourful photograph placed on the table. The time of a photograph exposition is unlimited and the photographs are scrolled by the experimenter. Body parts do not have to be named. When pointing to one’s own body or to the photograph, the side of the paired body part pointed to does not have to be kept. The experimenter measures the number of correct answers in both conditions separately (pointing to one’s own body and pointing to the photograph). Comparing this version of body parts localization task to the version which was used by Schwoebel and Coslett (2005), one will observe two modifications: 17 instead of 24 body parts are used, and participants are asked to point to a body part not only on their own body but also on the body in a photograph.

In the tactile input localization task, the participant is seated with his/her eyes shut while the experimenter touches various locations on his or her body with sticks. The locations include both odd (forehead, nose, abdomen, and neck) and paired body parts (hand, ear, foot, cheek, arm, and thigh). Each paired body part is touched separately on the left and right side of the body, as well as simultaneously on both sides (left and right at the same time), which amounts to a total of 22 tactile stimulations. Stimulations of the same body parts do not follow each other: they are separated by stimuli applied on other locations. After being touched, the participant is asked whether he/she had felt the touch. If he/she confirms, he/she is asked to point to the corresponding location on his/her own body and on the body in the picture. When pointing on their own body, the participant should keep the side of the stimulation unchanged. This is not required when a body part is shown in the picture. The number of correct answers in both conditions (pointing to one’s own body and pointing to the photograph) in total as well as for odd and paired body parts is measured separately. An answer is treated as correct when the participant is able to feel the touch but cannot point to its location or points to the location incorrectly. When the participant cannot feel the touch, he/she is also unable to point to its location. Such a situation is classified as “no error”. In relation to the original task used by Schwoebel and Coslett (2005), three modifications were made. Firstly, 22 instead of 20 locations are used. Secondly, participants are asked to point to body parts on their own body and on the body in a picture, not on a mannequin. Lastly, the question whether the participant felt the touch is posed to control possible tactile deficits.

In the task of matching body parts by location, the participant is shown sets of four photographs of body parts on the computer screen: one at the top and three at the bottom of each slide. After the exposition of each set, the participant is asked to decide which of the three lower body parts is the closest to the target body part shown above. Each participant is instructed that he/she should refer to body part locations in a typical standing position. To be sure that the participants have understood the instruction, a trial set precedes the scored ones. The assessment stage consists of 13 sets of photographs. In each set in the lower line of body parts, there is one correct answer and two distractors. One distractor is a body part that comes from the same body region and one is from a distant body area (e.g. for a knee presented at the top of the slide, there are three body parts at the bottom: an abdomen—a body part from a distant body area, a calf—the correct answer, and a foot—a body part of the same body region, but not the closest one). The slides are changed by the experimenter. The target body parts in the subsequent slides do not come from the same body regions. The order of correct answers is random. The experimenter should not name the presented body parts. The number of correct answers is measured. Compared to the study by Schwoebel and Coslett (2005), the number of sets in this task was reduced (13 instead of 24 sets are used).

#### Body semantics tasks

To assess body semantics, three tasks were used: designating body parts, naming body parts, and matching body parts to objects.

The designating body parts task consists of four slides with six (three slides) or four (one slide) photographs of body parts displayed on the computer screen. A total of 22 colourful photographs of body parts are used (foot, ear, elbow, neck, abdomen, thumb, nose, knee, hand, calf, forehead, toes, head, thigh, mouth, back, face, arm, eye, chest, eyebrow, and heel). The participant is instructed to point to the body part that is named by the experimenter. The number of correct answers is measured. The task was the author’s and was not used by Schwoebel and Coslett in their study (2005).

In the naming body parts task, the participant is instructed to give the names of isolated body parts, which are displayed individually on the computer screen. Twenty-two colourful photographs of body parts are used (the same as those in the designating body parts task). The experimenter measures the number of correct names as well as errors. This task was not used by Schwoebel and Coslett (2005).

Matching body parts to objects consists in choosing that of three body parts which is the most closely associated with the object displayed. A photograph of an object is shown at the top of each slide, and the three body parts are displayed below. Besides the correct answers, which appear in a random order, there are a contiguous body part (from the same body area as the correct answer) and an unrelated body part (from another body region). There is one trial set which is performed together with the experimenter to make sure that the participant can understand the instruction. At the assessment stage, ten colourful photographs of objects are used (glasses, watch, bag, earring, belt, shoe, lipstick, scarf, tissue, and hat). The recognition of the objects is checked by the tasks assessing language functions during general neuropsychological examination (the objects are included in “other accessories” category). The number of correct answers and the number of errors are measured in the task. Compared to the original task used by Schwoebel and Coslett (2005), fewer sets of photographs are used in this case (i.e. 10 instead of 20).

The task of matching body parts by function (see Schwoebel and Coslett 2005) was not included in the study, because of some methodological doubts. It was difficult, in the author’s opinion, to design a task comprising sets of body parts similar in terms of function where the number of trials would be comparable to those applied in other body representation tasks (Tables 6, 7, 8).

**Table 6:** Mean scores for the patients and healthy controls in general neuropsychological measures

	Patients after brain injuries, *M* (SD)	Healthy controls, *M* (SD)	Mann–Whitney *U*/*t* test	*p*
Attention—correct answers in total^a^	11.53 (11.10)	11.94 (0.25)	*U* = 959.00	.05
Attention—reaction time (ms)	1537 (909.92)	782.19 (213.66)	*t*(53.49) = 5.65 *d* = 0.99	.001
Designating objects—correct answers	34.86 (0.14)	35 (0.00)	*U* = 1175.00	*ns*
Naming objects—errors	2.36 (5.19)	0.00 (0.00)	*U* = 600.00	.001
Executing commands—correct answers	12.28 (1.70)	13.46 (1.15)	*U* = 723.00	.001
Working memory (Digit Span)	8.06 (1.82)	9.42 (1.98)	*U* = 809.00	.01
Visuo-spatial functions—correct answers	8.10 (1.31)	8.56 (0.64)	*U* = 1086.00	*ns*

*ms* milliseconds, *ns* not significant

^a^In this task, a patient with hemianopsia was excluded; she participated in other tasks, as she was able to compensate her deficit; because of technical problems, the healthy controls group in this task consisted of 47 participants

**Table 7:** Mean correct response time in the hand laterality task in both groups (in milliseconds)

	Patients after brain injuries, *M* (SD)	Healthy controls, *M* (SD)	Mann–Whitney *U*/*t* test	*p*
R hand	5070.36 (4309.40)	3207.48 (1741.46)	*U* = 772.00	.01
R hand palm-down	4655.88 (3926.92)	3115.52 (1596.77)	*U* = 827.00	.01
R hand palm-up	544,708 (4921.95)	3255.26 (2062.74)	*U* = 799.50	.01
L hand	4588.76 (2808.25)	3072.14 (1280.18)	*t*(68.52) = 3.46 *d* = 0.66	.001
L hand palm-down	4385.46 (2743.60)	3142.44 (1154.19)	*t*(65.82) = 2.95 *d* = 0.57	.01
L hand palm-up	4759.04 (3077.83)	3022.12 (1681.44)	*t*(75.86) = 3.50 *d* = 0.66	.001

*R* right, *L* left

**Table 8:** Mean time of movement performance in the hand action task (in seconds)

	Patients after brain injuries, *M* (SD)	Healthy controls, *n* = 50, *M* (SD)	Mann–Whitney *U*/*t* test	*p*
Right hand				
Eyes opened	*n* = 41		*U* = 92.00	.001
	3.00 (2.30)	1.05 (0.34)		
Eyes closed	*n* = 41		*U* = 108.00	.001
	3.23 (2.42)	1.13 (0.39)		
Left hand				
Eyes opened	*n* = 45		*t*(46.09) = 6.53	.001
	3.20 (2.16)	1.08 (0.35)	*d* = 1.15	
Eyes closed	*n* = 45		*t*(46.20) = 7.49	.001
	3.60 (2.16)	1.16 (0.36)	*d* = 1.26	

## Footnotes

1 Data were gathered through interview which might have been influenced by neuropsychological and self-awareness deficits; that is why the conclusion should be treated carefully.

## References

[CR1] Amorim M-A, Isableu B, Jarraya M (2006). Embodied spatial transformations: “body analogy” for the mental rotation of objects. J Exp Psychol Gen.

[CR2] Anema HA, van Zandvoort MJE, de Haan EHF (2009). A double dissociation between somatosensory processing for perception and action. Neuropsychologia.

[CR3] Barcikowska M, Bilikiewicz A (2004). Choroba Alzheimera w teorii i praktyce klinicznej.

[CR4] Botvinick M (2004). Probing the neural basis of body ownership. Science.

[CR5] Boyer TW, Maouene J, Sethuraman N (2017). Attention to body-parts varies with visual preference and verb-effector associations. Cogn Process.

[CR6] Bracco F, Chiorri C (2009). People have the power: priority of socially relevant stimuli in a change detection task. Cogn Process.

[CR7] Brytek-Matera A, Rybicka-Klimczyk A (2009). Wizerunek ciała w anoreksji i bulimii psychicznej.

[CR8] Brzeziński J, Gaul M, Hornowska E et al (2004) WAIS-R (PL) - Skala Inteligencji D. Wechslera dla Dorosłych - Wersja zrewidowana. Renormalizacja 2004. Pracownia Testów Psychologicznych Polskiego Towarzystwa Psychologicznego, Warszawa

[CR9] Burin D, Livelli A, Garbarini F (2015). Are movements necessary for the sense of body ownership? Evidence from the rubber hand illusion in pure hemiplegic patients. PLoS ONE.

[CR10] Buxbaum LJ, Coslett HB (2001). Specialised structural descriptions for human body parts: evidence from autotopagnosia. Cogn Neuropsychol.

[CR11] Chatterjee A (1996). Anosognosia for hemiplegia: patient retrospections. Cognit Neuropsychiatry.

[CR12] Churchland PS (2002). Self-representation in nervous systems. Science.

[CR13] Corradi-Dell’Acqua C, Rumiati RI, Santoianni F, Sabatano C (2007). What the brain knows about the body: evidence for dissociable representations. Brain development in learning environments: embodied and perceptual advancements.

[CR14] Coslett HB (1998). Evidence for a disturbance of the body schema in neglect. Brain Cogn.

[CR15] Creem-Regehr SH, Neil JA, Yeh HJ (2007). Neural correlates of two imagined egocentric transformations. Neuroimage.

[CR16] Cuthbert SC, Goodheart GJ (2007). On the reliability and validity of manual muscle testing: a literature review. Chiropr Osteopat.

[CR17] Denes G, Cappelletti JY, Zilli T (2000). A category-specific deficit of spatial representation: the case of autotopagnosia. Neuropsychologia.

[CR18] Felician O, Ceccaldi M, Didic M (2003). Pointing to body parts: a double dissociation study. Neuropsychologia.

[CR19] Galati G, Committeri G, Sanes JN, Pizzamiglio L (2001). Spatial coding of visual and somatic sensory information in body-centred coordinates. Eur J Neurosci.

[CR20] Gallagher S (2006). How the body shapes the mind.

[CR21] Gallagher S, Cole J (1995). Body image and body schema in a deafferented Subject. J Mind Behav.

[CR22] Gerardin E, Sirigu A, Lehéricy S (2000). Partially overlapping neural networks for real and imagined hand movements. Cereb Cortex.

[CR23] Giummarra MJ, Gibson SJ, Georgiou-Karistianis N, Bradshaw JL (2008). Mechanisms underlying embodiment, disembodiment and loss of embodiment. Neurosci Biobehav Rev.

[CR24] Guariglia C, Piccardi L, Puglisi Allegra MC, Traballesi M (2002). Is autotopoagnosia real? EC says yes. A case study. Neuropsychologia.

[CR25] Haggard P, Taylor-Clarke M, Kennett S (2003). Tactile perception, cortical representation and the bodily self. Curr Biol CB.

[CR26] Holmes NP, Spence C (2004). The body schema and the multisensory representation(s) of peripersonal space. Cogn Process.

[CR27] Holmes NP, Spence C, Knoblich G, Thornton IM, Grosjean M, Shiffrar M (2006). Beyond the body schema: visual, prosthetic, and technological contributions to bodily perception and awareness. Human body perception from the inside out: advances in visual cognition.

[CR28] Jeannerod M (1999). The 25th Bartlett Lecture. To act or not to act: perspectives on the representation of actions. Q J Exp Psychol Sect A.

[CR29] Jeannerod M (2004). Visual and action cues contribute to the self-other distinction. Nat Neurosci.

[CR30] Kaplan E, Goodglass H, Weintraub S (1983). Boston naming test.

[CR31] Kemmerer D, Tranel D (2008). Searching for the elusive neural substrates of body part terms: a neuropsychological study. Cogn Neuropsychol.

[CR32] Laiacona M, Allamano N, Lorenzi L, Capitani E (2006). A case of impaired naming and knowledge of body parts. Are limbs a separate sub-category?. Neurocase.

[CR33] Llorens R, Borrego A, Palomo P (2017). Body schema plasticity after stroke: subjective and neurophysiological correlates of the rubber hand illusion. Neuropsychologia.

[CR34] Longo MR, Azañón E, Haggard P (2010). More than skin deep: body representation beyond primary somatosensory cortex. Neuropsychologia.

[CR35] Maravita A, Knoblich G, Thornton I, Grosjean M, Shiffrar M (2006). From “body in the brain” to “body in space”. Sensory and intentional components of body representation. Human body perception from the inside out.

[CR36] Maselli A (2015). Allocentric and egocentric manipulations of the sense of self-location in full-body illusions and their relation with the sense of body ownership. Cogn Process.

[CR37] Medina J, Coslett HB (2010). From maps to form to space: touch and the body schema. Neuropsychologia.

[CR38] Mohr C, Blanke O, Brugger P (2006). Perceptual aberrations impair mental own-body transformations. Behav Neurosci.

[CR39] Mozaz MJ, Morris RG (1997). Identification of body parts in Alzheimer’s disease: evidence for a body schema hypothesis. Int J Neurosci.

[CR40] Ogden JA (1985). Autotopagnosia. Occurrence in a patient without nominal aphasia and with an intact ability to point to parts of animals and objects. Brain. J Neurol.

[CR41] Paillard J, Gantchev GN, Mori S, Massion J (1999). Body schema and body image—a double dissociation in deafferented patients. Motor control, today and tomorrow.

[CR42] Parsons LM (1987). Imagined spatial transformation of one’s body. J Exp Psychol Gen.

[CR43] Press C, Heyes C, Haggard P, Eimer M (2008). Visuotactile learning and body representation: an ERP study with rubber hands and rubber objects. J Cogn Neurosci.

[CR44] Reed CL, Farah MJ (1995). The psychological reality of the body schema: a test with normal participants. J Exp Psychol Hum Percept Perform.

[CR45] Reisberg B, Ferris SH, de Leon MJ, Crook T (1988). Global Deterioration Scale (GDS). Psychopharmacol Bull.

[CR46] Rizzolatti G, Fogassi L, Gallese V (1997). Parietal cortex: from sight to action. Curr Opin Neurobiol.

[CR47] Ro T, Friggel A, Lavie N (2007). Attentional biases for faces and body parts. Vis Cogn.

[CR48] Schwoebel J, Coslett HB (2005). Evidence for multiple, distinct representations of the human body. J Cogn Neurosci.

[CR49] Schwoebel J, Friedman R, Duda N, Coslett HB (2001). Pain and the body schema: evidence for peripheral effects on mental representations of movement. Brain J Neurol.

[CR50] Schwoebel J, Boronat CB, Branch Coslett H (2002). The man who executed “imagined” movements: evidence for dissociable components of the body schema. Brain Cogn.

[CR51] Schwoebel J, Buxbaum LJ, Coslett HB (2004). Representations of the human body in the production and imitation of complex movements. Cogn Neuropsychol.

[CR52] Sekiyama K (2006). Dynamic spatial cognition: components, functions, and modifiability of body schema. Jpn Psychol Res.

[CR53] Semenza C (1988). Impairment in localization of body parts following brain damage. Cortex.

[CR54] Semenza C, Delazer M, Code C, Wallesch C-W, Joanette Y, Lecours AR (2003). Pick’s cases on body representation (1908, 1915, 1922): a retrospective assessment. Classic cases in neuropsychology.

[CR55] Semenza C, Goodglass H (1985). Localization of body parts in brain injured subjects. Neuropsychologia.

[CR56] Shontz FC, McNish RD (1972). The human body as stimulus object: estimates of distances between body landmarks. J Exp Psychol.

[CR57] Sirigu A, Grafman J, Bressler K, Sunderland T (1991). Multiple representations contribute to body knowledge processing. Evidence from a case of autotopagnosia. Brain. J Neurol.

[CR58] Stephan KM, Fink GR, Passingham RE (1995). Functional anatomy of the mental representation of upper extremity movements in healthy subjects. J Neurophysiol.

[CR59] Suzuki K, Yamadori A, Fuji T (1997). Category-specific comprehension deficit restricted to body parts. Neurocase.

[CR60] Tagini A, Raffone A (2010). The “I” and the “Me” in self-referential awareness: a neurocognitive hypothesis. Cogn Process.

[CR61] Tsakiris M, Hesse MD, Boy C (2007). Neural signatures of body ownership: a sensory network for bodily self-consciousness. Cereb Cortex.

[CR62] Vallar G, Papagno C, Code C, Wallesch C-W, Joanette Y, Lecours AR (2003). Pierre Bonnier’s (1905) cases of bodily “aschematie”. Classic cases in neuropsychology.

[CR63] van Stralen HE, van Zandvoort MJE, Kappelle LJ, Dijkerman HC (2013). The rubber hand illusion in a patient with hand disownership. Perception.

[CR64] Wolak K (1989). Problematyka badań nad tożsamością własnej cielesności. Przegląd Psychol.

[CR65] Zimmerman-Górska I, Gajewski P (2012). Choroby reumatyczne. Objawy podmiotowe i przedmiotowe. Interna Szczeklika: podręcznik chorób wewnętrznych 2012.

